# Beyond cut-offs: gestational age-specific perinatal mortality across the birthweight-for-gestational-age continuum—a population-based cross-sectional study

**DOI:** 10.1007/s00431-026-07233-6

**Published:** 2026-07-15

**Authors:** Liset Hoftiezer, Michel H. P. Hof, Richard A. van Lingen, Chantal W. P. M. Hukkelhoven, Marije Hogeveen

**Affiliations:** 1 Department of Neonatology, Amalia Children’s Hospital, Radboudumc Graduate School, Radboud University Medical Centre, Nijmegen, The Netherlands; 2 Department of Neonatology, Princess Amalia Department of Pediatrics, Isala, Zwolle, The Netherlands; 3 Department of Pediatrics, Slingeland Hospital, Doetinchem, The Netherlands; 4 Department of Epidemiology and Data, Amsterdam UMC Location University of Amsterdam, Amsterdam, The Netherlands; 5 Perined, Utrecht, The Netherlands

**Keywords:** Birthweight, Percentiles, SGA, Perinatal mortality

## Abstract

Abnormal fetal growth is linked to increased perinatal morbidity and mortality. Birthweight percentiles—commonly the 10th and 90th—define small (SGA) and large for gestational age (LGA) infants. Although birthweight charts are periodically updated, cut-offs for SGA and LGA remain fixed, despite changes in population characteristics and evolving clinical management. We aimed to characterize how perinatal mortality varies continuously across birthweight-for-gestational-age (BW-for-GA) and gestational age (GA), describing GA-specific mortality gradients rather than defining new cut-offs. We analyzed 2,581,816 singleton infants born in the Netherlands (2000–2015), excluding congenital malformations, extreme birthweights, or missing data. Perinatal mortality was defined as fetal or early neonatal death (< 7 days). Logistic regression modeled mortality based on continuous GA and BW-for-GA percentile, with rate ratios calculated relative to the 50th percentile and GA-specific nadir. Sensitivity analyses excluded antepartum stillbirths. Perinatal mortality declined gradually with GA, reaching a nadir at 40 weeks. Across BW-for-GA, mortality was lowest at the 80th–83rd percentile and higher at both extremes. Infants below the 3rd percentile had at least a twofold increase in mortality compared with those at median birthweight, while comparable increases for infants below the 10th percentile were observed only after 37 weeks. Sensitivity analyses excluding antepartum stillbirths reduced overall mortality but preserved the curvilinear pattern.

*Conclusion*: Perinatal mortality varies continuously, without discrete inflection points, revealing graded, gestational-age-specific patterns across the population and highlighting the limitations of rigid SGA and LGA thresholds.

**Table:** 

What is Known: • *SGA and LGA infants face higher risks of adverse outcomes.* • *Although birthweight charts are regularly updated, fixed percentile cut-offs such as SGA and LGA are rarely reconsidered despite changing populations and evolving clinical management.*	
What is New: • *Perinatal mortality varies continuously across birthweight-for-gestational-age, without discrete inflection points, revealing graded, GA-specific patterns.* • *This highlights the limitations of fixed SGA/LGA percentile thresholds and supports nuanced interpretation of risk for clinical decision-making.*

**Supplementary Information:**

The online version contains supplementary material available at 10.1007/s00431-026-07233-6.

## Introduction

Deviation of normal fetal growth is associated with increased perinatal morbidity and mortality [1, 2]. Birthweight, as the ultimate measurable result of fetal growth, is often used retrospectively to identify infants who may have failed to achieve their genetic growth potential. Although its causal role in mortality remains debated, birthweight serves as a convenient surrogate for other factors associated with adverse perinatal outcomes [3]. Historically, low birthweight was defined as < 2500 g, a threshold first proposed in 1919 without a clear biological rationale, and for a long time synonymous with prematurity [4]. In the 1960 s, a more nuanced distinction between low birthweight and prematurity led to the introduction of small, appropriate and large for gestational age (SGA, AGA, LGA) classifications [5, 6]. Today, most researchers define SGA as < 10th percentile and LGA as > 90th percentile on birthweight or fetal weight charts [3, 7].

Despite their descriptive nature, the 10th and 90th percentiles are frequently used to guide clinical decisions, such as initiating glucose monitoring, although evidence for predicting individual risk is limited. The main advantage of dichotomizing birthweight is its simplicity, making results easier to present, understand, and apply in clinical practice. A major disadvantage is information loss: infants close to but on opposite sides of the 10th or 90th percentile are seen as very different rather than very similar. Likewise, all AGA infants are treated as equivalent, whether their birthweight was at the 11th or the 89th percentile or anywhere in between. Furthermore, it is assumed that the 10th and 90th percentiles are equally valid to assign a risk status at 24 + 0 weeks’ as they are at 42 + 0 weeks’ gestation. Finally, the assumption is made that the cut-offs are relevant for multiple distinct outcome measures.

Although birthweight charts are regularly updated, the thresholds used to define abnormal birthweight are rarely reconsidered. The 10th percentile continues to define small for gestational age (SGA), even though differences in chart assumptions can substantially shift this threshold [8]. As a result, different infants may be classified as SGA depending on the chart used and may subsequently undergo management based on that classification, such as glucose monitoring.

Previous studies have suggested that perinatal risk varies across the birthweight distribution, with the lowest mortality observed above the 80th percentile, highlighting a curvilinear, gestational age (GA)–dependent relationship [9, 10]. However, detailed data on perinatal mortality across specific birthweight percentiles and GA remain limited. Therefore, the aim of our study was to describe how perinatal mortality varies across the continuous distribution of birthweight-for-gestational-age (BW-for-GA) and how this relationship depends on GA, using national registry data. Our primary objective was not to define new cut-offs, but to provide a detailed, descriptive illustration of GA-specific mortality gradients across the full birthweight distribution. By examining these continuous patterns, we highlight the limitations of dichotomous definitions (SGA/LGA) in capturing the nuances of risk.

## Methods

### Setting

Data were extracted from the Dutch perinatal registry (Perined), which is a linked database of medical registries from the four professional organizations that provide perinatal care in The Netherlands [11]. The database includes both home deliveries and hospital deliveries. In over 95% of the pregnancies GA is certain, either confirmed by or based on early ultrasound. The registry contains detailed anonymized information on pregnancies, deliveries, and neonatal (re)admissions. Overall, the database contains data of 97% of all pregnancies in The Netherlands and is considered an unbiased representation of the Dutch population [12].

### Participants

For the present analysis, we used data on births between January 1, 2000 and December 31, 2015. We restricted the study to singleton births at 24–42 weeks gestation without any reported congenital malformations. Records with missing gender, birthweight, or GA were excluded. Infants born < 25 + 0 weeks gestation were included from 2011 onwards, when the national Dutch guideline on perinatal practice in extremely premature delivery lowered the limit offering intensive care from 25 + 0 to 24 + 0 weeks gestation [13]. Prior to the implementation of this guideline, infants born < 25 + 0 weeks gestation were unlikely to survive because life-sustaining treatment was withheld.

For each infant, the birthweight percentile was calculated using the national birthweight charts [14]. Infants with extreme birthweights, defined as below the 0.1th or above the 99.9th percentile, were considered unrealistic and excluded from the analysis.

### Variables

Our primary outcome of interest, perinatal mortality, was defined as fetal death prior to or during labor or early neonatal death within 7 days after birth [15]. We examined the association between perinatal mortality, GA, and birthweight percentile using logistic regression. Birthweight percentiles were derived from an established reference chart [14] we previously developed and that has been nationally implemented; it is based on a Box–Cox t distribution, which accounts for skewness and kurtosis in birthweight. Because this distribution does not permit simple conversion to z-scores, modelling on the percentile scale is most consistent with the reference; alternative parameterizations were not used, as they would not alter the characterization of the risk gradient, which is the primary focus rather than specific numeric cut-offs.

### Statistical analysis

Percentiles are inherently bounded (0–100) and nonlinear, so we treated them as continuous to describe the shape and gradient of mortality risk across the distribution rather than to define thresholds. This descriptive approach characterizes population-level patterns rather than individual predictions. The relation between perinatal mortality and GA and birthweight was modelled with the following logistic regression model:

$$\mathrm{log}\left[\frac{\mathit{Pr}\left(\mathrm{p}\mathrm{e}\mathrm{r}\mathrm{i}\mathrm{n}\mathrm{a}\mathrm{t}\mathrm{a}\mathrm{l}\; \mathrm{m}\mathrm{o}\mathrm{r}\mathrm{t}\mathrm{a}\mathrm{l}\mathrm{i}\mathrm{t}\mathrm{y}\right)}{1-\mathit{Pr}\left(\mathrm{p}\mathrm{e}\mathrm{r}\mathrm{i}\mathrm{n}\mathrm{a}\mathrm{t}\mathrm{a}\mathrm{l}\; \mathrm{m}\mathrm{o}\mathrm{r}\mathrm{t}\mathrm{a}\mathrm{l}\mathrm{i}\mathrm{t}\mathrm{y}\right)}\right]=\alpha +{f}_{1,{\lambda}_{1}}\left(\mathrm{g}\mathrm{e}\mathrm{s}\mathrm{t}\mathrm{a}\mathrm{t}\mathrm{i}\mathrm{o}\mathrm{n}\mathrm{a}\mathrm{l}\; \mathrm{a}\mathrm{g}\mathrm{e}\right)+ {f}_{2,{\lambda}_{2}}\left(\mathrm{b}\mathrm{i}\mathrm{r}\mathrm{t}\mathrm{h}\mathrm{w}\mathrm{e}\mathrm{i}\mathrm{g}\mathrm{h}\mathrm{t}\; \mathrm{p}\mathrm{e}\mathrm{r}\mathrm{c}\mathrm{e}\mathrm{n}\mathrm{t}\mathrm{i}\mathrm{l}\mathrm{e}\right)+ {f}_{3,{\lambda}_{3}}\left(\mathrm{g}\mathrm{e}\mathrm{s}\mathrm{t}\mathrm{a}\mathrm{t}\mathrm{i}\mathrm{o}\mathrm{n}\mathrm{a}\mathrm{l}\; \mathrm{a}\mathrm{g}\mathrm{e}, \mathrm{b}\mathrm{i}\mathrm{r}\mathrm{t}\mathrm{h}\mathrm{w}\mathrm{e}\mathrm{i}\mathrm{g}\mathrm{h}\mathrm{t}\; \mathrm{p}\mathrm{e}\mathrm{r}\mathrm{c}\mathrm{e}\mathrm{n}\mathrm{t}\mathrm{i}\mathrm{l}\mathrm{e}\right),$$

Where ${f}_{1,{\lambda}_{1}}\left(x\right)$ and d ${f}_{2,{\lambda}_{2}}\left(z\right)$ are smooth functions based on cubic splines. The cubic spline functions are constructed using one hundred knot points that are equally spaced over the entire range of observed values. The level of smoothness is controlled by the penalty parameters ${\lambda}_{1}$ and ${\lambda}_{2}$. In addition, the function ${f}_{3{\lambda}_{3}}\left(x, z\right)$ is a smooth function based on a tensor product of cubic splines. For this function, we used ten equally spaced knots in each dimension and the level of smoothness was controlled by the penalty parameter and ${\lambda}_{3}$. The interaction was included to allow the risk-percentile relationship to vary by GA (non-parallel risk gradients across gestation).

After fitting the logistic regression model, the estimates were used to calculate rate ratios for selected birthweight percentiles relative to both the 50th percentile (i.e., median) and the gestational-age-specific nadir, defined as the lowest mortality observed at each GA. Corresponding 95% confidence intervals of these rate ratios were obtained using posterior inference [16]. The primary analysis included all perinatal deaths, while the sensitivity analysis excluded antepartum stillbirths to reduce bias from in-utero weight loss and uncertainty in timing of death.

All statistical analyses were performed with R 4.3.0 (R Core Team (2017), R Foundation for Statistical Computing, Vienna, Austria). The R package mgcv was used for the logistic regression analysis and the subsequent analysis of the rate ratios based on posterior inference [17].

## Results

**Fig. 1:**
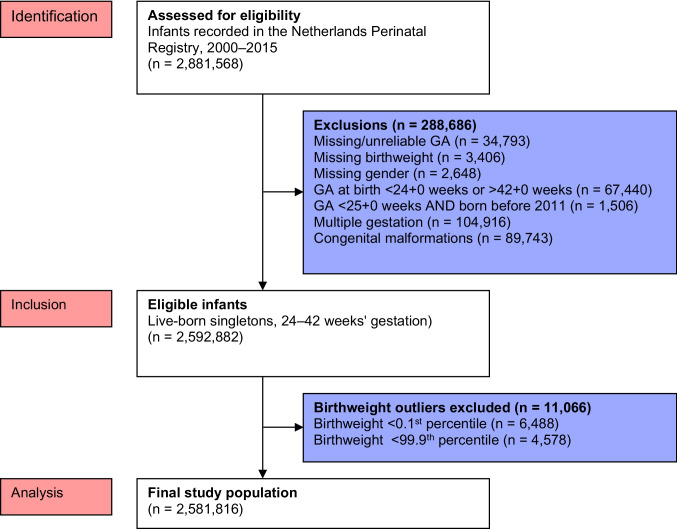
Flow diagram of study participants

Between 2000 and 2015, almost 2.9 million infants were born in The Netherlands and recorded in our national registry. We excluded 288,686 infants (10.0%) who fulfilled our exclusion criteria (Fig. 1). Another 11,066 infants (0.4%) were excluded because of unrealistically low or high birthweights for their GA. The final study population consisted of 2,581,816 singleton infants. Baseline characteristics are shown in Supplementary File 1. Among the excluded infants were relatively more preterm and/or low birthweight infants as well as hospital births. Other differences were small and not clinically relevant, albeit presumably statistically significant due to extremely large sample sizes. For the sensitivity analyses, 7965 antepartum stillbirths were excluded.

Between 2000 and 2015, 2,881,568 infants were recorded in the Netherlands Perinatal Registry. After excluding 288,686 infants who met one or more exclusion criteria and 11,066 birthweight outliers (< 0.1st or > 99.9th percentile according to Hoftiezer et al. [14]), the final study population comprised 2,581,816 infants.

Table 1 shows the distribution of fetal and neonatal mortality in the study population. The overall perinatal mortality rate (i.e., per 1000 births) was 4.4, ranging from 1.5 in term infants born at 40 weeks gestation to 627.7 in extremely preterm infants born at 24 weeks gestation, and from 2.2 in infants with birthweights between the 50th and < 90th percentile to 29.7 in infants with birthweights below the 3rd percentile.

**Table 1:** Distribution of fetal and neonatal mortality by gestational age and birthweight percentile

GA, completed weeks^a^								
				Fetal death		Neonatal death		
	Total births (*n* [cumulative %])		Total deaths (*n* [%])	Antepartum stillbirth (*n* [%])	Intrapartum death (*n* [%])	Death < 24 h after birth (*n* [%])	Death 2–7 days (*n* [%])	Death 8–28 days (*n* [%])^b^
24	411 [0.02]		280 [68.13]	100 [24.33]	39 [9.49]	75 [18.25]	44 [10.71]	22 [5.35]
25	1422 [0.07]		909 [63.92]	455 [32.00]	74 [5.20]	223 [15.68]	76 [5.34]	81 [5.70]
26	1755 [0.14]		736 [41.94]	440 [25.07]	39 [2.22]	83 [4.73]	79 [4.50]	95 [5.41]
27	2035 [0.22]		619 [30.42]	388 [19.07]	21 [1.03]	50 [2.46]	86 [4.23]	74 [3.64]
28	2332 [0.31]		504 [21.61]	332 [14.24]	23 [0.99]	44 [1.89]	49 [1.89]	56 [2.40]
29	2800 [0.42]		436 [15.57]	317 [11.32]	20 [0.71]	30 [1.07]	41 [1.07]	28 [1.00]
30	3747 [0.56]		437 [11.66]	332 [8.86]	21 [0.56]	34 [0.91]	34 [0.91]	16 [0.43]
31	4837 [0.75]		408 [8.43]	309 [6.39]	25 [0.52]	30 [0.62]	31 [0.64]	13 [0.27]
32	7093 [1.02]		432 [6.09]	346 [4.88]	22 [0.31]	32 [0.45]	27 [0.38]	5 [0.07]
33	10,841 [1.44]		434 [4.00]	346 [3.19]	22 [0.20]	38 [0.35]	22 [0.20]	6 [0.06]
34	18,625 [2.17]		493 [2.65]	404 [2.17]	21 [0.11]	43 [0.23]	22 [0.12]	3 [0.02]
35	30,524 [3.35]		537 [1.76]	427 [1.40]	31 [0.10]	43 [0.14]	25 [0.08]	11 [0.04]
36	60,292 [5.68]		632 [1.05]	503 [0.83]	34 [0.06]	39 [0.06]	50 [0.08]	6 [0.01]
37	159,481 [11.86]		821 [0.51]	619 [0.39]	70 [0.04]	62 [0.04]	58 [0.04]	12 [0.01]
38	386,796 [26.84]		1024 [0.26]	736 [0.19]	94 [0.02]	75 [0.02]	98 [0.03]	21 [0.01]
39	634,427 [51.41]		1114 [0.18]	717 [0.11]	141 [0.02]	98 [0.02]	134 [0.02]	24 [0.00]
40	737,568 [79.98]		1129 [0.15]	681 [0.09]	151 [0.02]	117 [0.02]	157 [0.02]	23 [0.00]
41	471,808 [98.26]		897 [0.19]	479 [0.10]	156 [0.03]	104 [0.02]	135 [0.03]	23 [0.00]
42	45,022 [100.00]		85 [0.19]	34 [0.08]	20 [0.04]	13 [0.03]	11 [0.02]	7 [0.02]
Total	2,581,816		11,927 [0.46]	7,965 [0.31]	1,024 [0.04]	1,233 [0.05]	1,179 [0.05]	526 [0.02]
Birthweight percentile								
				Fetal death		Neonatal death		
	Total births (*n* [cumulative %])	Total deaths (*n* [%])		Antepartum stillbirth (*n* [%])	Intrapartum death (*n* [%])	Death < 24 h after birth (*n* [%])	Death 2–7 days (*n* [%])	Death 8–28 days (*n* [%])^b^
< 3rd	102,124 [3.96]	3154 [3.09]		2,446 [2.40]	196 [0.19]	184 [0.18]	202 [0.20]	126 [0.12]
3– < 5th	57,346 [6.18]	720 [1.26]		535 [0.93]	46 [0.08]	57 [0.10]	57 [0.10]	25 [0.04]
5– < 10th	136,067 [11.45]	1056 [0.78]		720 [0.53]	109 [0.08]	98 [0.07]	96 [0.07]	33 [0.02]
10– < 50th	1,007,750 [50.48]	3858 [0.38]		2450 [0.24]	350 [0.03]	444 [0.04]	431 [0.04]	183 [0.02]
50– < 90th	1,000,768 [89.24]	2332 [0.23]		1328 [0.13]	236 [0.02]	339 [0.03]	305 [0.03]	124 [0.01]
90– < 95th	133,293 [94.40]	329 [0.25]		183 [0.14]	34 [0.03]	47 [0.04]	42 [0.03]	23 [0.02]
95– < 97th	55,907 [96.57]	150 [0.27]		79 [0.14]	19 [0.03]	24 [0.04]	22 [0.04]	6 [0.01]
≥ 97th	88,561 [100.00]	328 [0.37]		224 [0.25]	34 [0.04]	40 [0.05]	24 [0.04]	6 [0.01]
Total	2,581,816	11,927 [0.46]		7965 [0.31]	1024 [0.04]	1233 [0.05]	1179 [0.05]	526 [0.02]

^a^GA in completed weeks refers to intervals of 24 + 0–24 + 6 weeks, 25 + 0–25 + 6 weeks, etc

^b^Neonatal deaths occurring after 7 days of life were not included in the definition of perinatal mortality

**Fig. 2:**
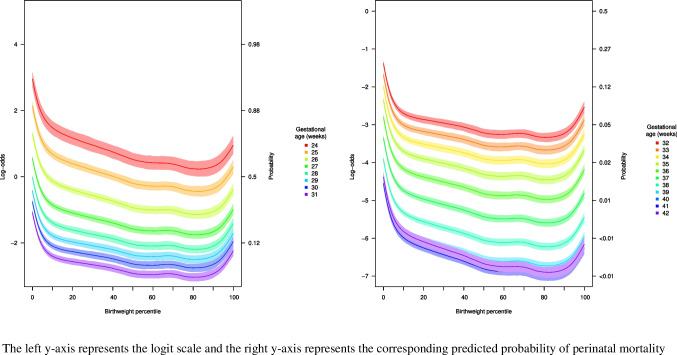
Gestational age-specific predicted probability of perinatal mortality across the birthweight-for-gestational-age continuum

Supplementary File 2 shows the individual effects of the explanatory variables. Gestational age had a clear effect, with mortality decreasing as gestation progressed and rising slightly after 285 days (40 + 6 weeks). The effect of birthweight percentile was smaller, with the highest mortality observed at the lower extremes of the distribution. Figure 2 illustrates absolute perinatal mortality across birthweight percentiles and GA. Overall, mortality declined with increasing GA, reaching its lowest point at 40 weeks, with the largest relative reduction occurring between 36 and 38 weeks, amounting to nearly a 50% decrease per week. Mortality followed a curvilinear pattern across birthweight percentiles, decreasing at higher percentiles and rising again beyond the 80th–83rd percentile. Absolute mortality remained substantially lower among the largest infants than in small infants.

**Fig. 3:**
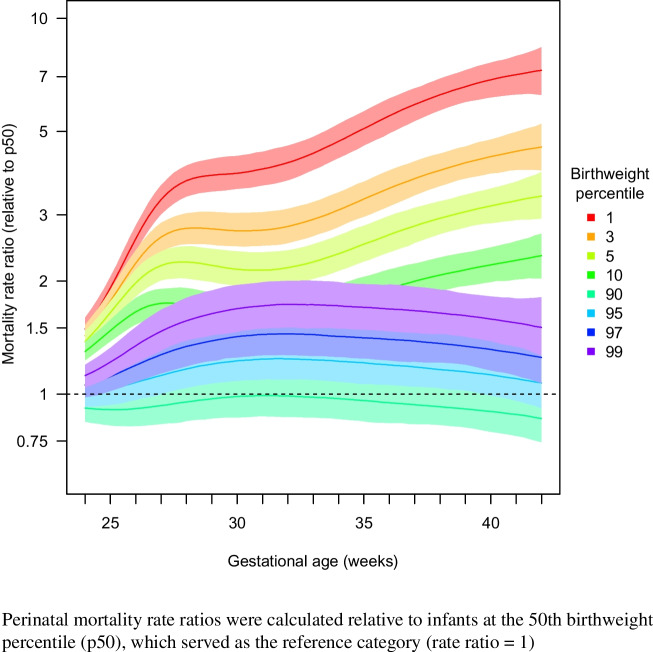
Perinatal mortality rate ratios relative to the 50th birthweight percentile (p50)

Figure 3 shows perinatal mortality rate ratios across birthweight percentiles relative to infants at the median, highlighting relative differences rather than absolute rates.

**Fig. 4:**
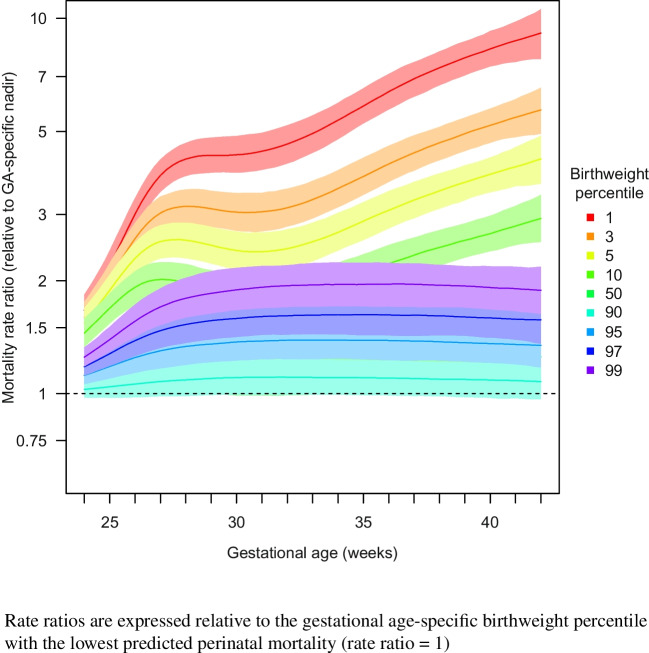
Perinatal mortality rate ratios relative to the GA-specific nadir

Except for infants born at 24–25 weeks, those below the 3rd percentile had approximately a twofold higher mortality rate compared with the median. From 37 weeks onward, infants at the 10th percentile also exhibited a twofold increase. When the GA–specific nadir was used as the reference group, the 10th percentile corresponded to approximately a twofold higher mortality rate beginning at 26 weeks (Fig. 4).

The highest rate ratio was observed among the smallest infants (1st percentile) at 42 weeks, with a value of 7.3 relative to the median and 9.1 relative to the GA–specific nadir (Figs. 3 and 4). At 42 weeks, even the 25th percentile identified infants with at least a twofold higher mortality rate compared with the GA–specific nadir (results not shown). At the upper end of the distribution, only infants above the 99th percentile after 29 weeks had a twofold or higher mortality rate relative to the GA–specific nadir (Fig. 4).

In a sensitivity analysis excluding antepartum stillbirths, overall perinatal mortality was reduced; however, the curvilinear pattern across birthweight percentiles persisted, though less prominently (Supplementary File 3). These results support the robustness of our finding that mortality rates vary continuously across GA and birthweight percentiles.

## Discussion

Birthweight at birth is inherently retrospective and therefore primarily informs postnatal surveillance and counselling rather than antenatal triage. This study was not designed to redefine clinical thresholds, but to characterize how perinatal mortality varies across the continuous distributions of BW-for-GA and GA, supporting population-level interpretation and communication of graded mortality patterns. We found that perinatal mortality does not show discrete shifts at conventional SGA and LGA cutoffs, challenging the often implicit assumption that dichotomous thresholds reflect abrupt changes in outcome. While infants at the extremes of birthweight experience higher mortality, these patterns are continuous and GA–dependent. Continuous, GA–specific analyses reveal a smooth mortality gradient across the full birthweight distribution, with a consistent nadir around the 80th–83rd percentile. Although the results pertain specifically to perinatal mortality, they may generate hypotheses for other neonatal or long-term outcomes that warrant further study.

Our results are in line with previous studies. Vasak et al. found the lowest perinatal mortality between the 80th and 90th percentiles [9], and another study reported the lowest mortality around 1SD above the mean (≈84th percentile), despite increases in mean birthweight and decreases in absolute mortality rate over time [18]. In very preterm births, weight between the median and 85th percentile predicted the highest survival [19]. For risk stratification, the focus is generally on populations at increased risk. Consistent with other studies reporting relative risk, we observed the highest perinatal mortality rates in the smallest infants [10, 20, 21] Boulet et al. highlighted that defining SGA without reference to gestational-age-specific mortality is limited; they developed risk curves based on birthweights associated with 2-, 2.5-, and threefold increased neonatal death compared with infants in the 45th–55th percentile [22]. In our study, the 10th percentile did not represent a distinct inflection point in mortality. When infants with median birthweight were used as the reference population, the 10th percentile corresponded to a twofold increase in mortality only in term infants. When compared with the gestational-age-specific nadir of mortality (80th–83rd percentile), the 10th percentile was associated with an approximately twofold increase in perinatal mortality from 26 weeks’ gestation onward; however, similar elevations were observed across several adjacent percentiles, extending up to approximately the 25th percentile depending on GA (results not shown). These findings underscore that the perceived risk with any given percentile is strongly influenced by both the choice of reference point and the characteristics of the reference population, highlighting the need for careful interpretation of percentile-based thresholds in perinatal risk stratification.

Absolute and relative measures of mortality provide complementary perspectives on clinical relevance. While rate ratios were highest in (post-)term infants, the absolute burden of being SGA was much greater in preterm infants. When the baseline mortality rate is high, even a rate ratio close to 1 may reflect a clinically meaningful effect, whereas large rate ratios may not always translate into significant outcomes. Clinical relevance also depends on the feasibility of preventing the adverse outcome, determined not only by the potential effectiveness of an intervention, but also by available resources and clinical capacity. For example, mortality rates increased after 39–40 weeks, particularly among fetuses with abnormal growth; yet such growth abnormalities may be difficult to identify in late pregnancy [23]. In several countries, there is a growing trend toward offering elective induction of labor at 39 weeks to mitigate this risk [24]. Nevertheless, despite substantial increases in induction rates, population-level stillbirth and perinatal mortality rates have not shown clear improvement [25].

Our primary outcome, perinatal mortality, includes stillbirths, intrapartum fetal deaths, and early neonatal deaths, which is why we refer to rate rather than risk. Estimating perinatal mortality risk by GA and birthweight percentile is not feasible, as fetal weight is unknown until birth [26]. Size at birth serves as a retrospective proxy for fetal growth, with clinical utility mainly for population surveillance and counselling rather than antenatal triage, which relies on estimated fetal weight-based charts and multiple risk factors. Most deceased infants (69.9%) were antepartum stillbirths, for which birthweight and GA may be unreliable due to the timing of fetal demise and the degree of maceration. Fetal weight may decrease in utero over time [27], and GA at delivery is influenced by the timing of fetal death, potentially leading to misclassification of growth status. To assess the impact of these issues, we repeated analyses excluding antepartum stillbirths. The overall patterns remained consistent, confirming that our main conclusions are robust and not materially affected by these limitations.

We observed the highest mortality rates at both extremes of the birthweight percentile distribution, though mechanisms leading to adverse outcomes in LGA infants (e.g., shoulder dystocia, cesarean delivery) likely differ from those affecting SGA infants. While adverse outcomes in term LGA infants are often linked to macrosomia (> 4000 g) [28] and labor complications, we observed twofold increased rate ratios in LGA infants as early as 29 weeks’ gestation. At this gestation, the 99th percentile corresponds to 1842 g for boys and 1781 g for girls [14], suggesting that birthweight alone does not explain the elevated mortality. Similarly, Baer et al. reported increased neonatal mortality in preterm LGA infants (28–31 weeks), independent of maternal diabetes (adjusted RR 2.1; 95% CI 1.5–2.9) [29]. These observations suggest that preterm LGA infants may face distinct risks that warrant further investigation.

### Strengths and limitations

The principal strength of our study was the use of a very large, nationwide cohort of infants to investigate the distribution of perinatal mortality. Gestational age and birthweight percentile were analyzed as continuous variables, avoiding dichotomization or categorization into strata [7, 9, 10, 20]. To facilitate a nuanced interpretation and understanding of clinical relevance, we reported both absolute and relative measures of perinatal mortality. Additionally, a sensitivity analysis confirmed the robustness of our findings.

We acknowledge several limitations, most inherent to our retrospective design. Extreme outliers with implausible combinations of GA and birthweight were excluded. Their perinatal mortality rate was more than 20 times higher than the study population and was not limited to antepartum stillbirths, suggesting that some may reflect true pathology rather than random data errors [30]. Excluding these deaths may have led to underestimation of mortality at the extremes of the birthweight distribution. Birthweight at birth is an imperfect proxy for fetal growth, and residual confounding, management decisions around viability thresholds, and possible registry misclassification may have influenced gestational-age-specific patterns. Our database lacked sufficient clinical detail to determine causes of death, so we could not assign specific causes, potentially overestimating absolute perinatal mortality attributed to suboptimal growth. Nevertheless, there is little reason to suspect that mortality rate ratios were differentially affected, as all-cause mortality was included in both the numerator and denominator [22]. Finally, our data were collected between 2000 and 2015, during which the overall perinatal mortality rate decreased from 5.9 in 2000 to 3.1 in 2015. Temporal trends had minimal effect on rate ratios (results not shown) [18]. Importantly, our findings are descriptive and should not be interpreted as estimates of individual risk. Absolute mortality rates should be interpreted with caution in light of these considerations.

### Generalizability

Worldwide, birthweight charts are updated periodically, yet, despite major differences in methodology, the thresholds to define abnormal birthweight are rarely questioned. Although infants at the extremes of the birthweight distribution do have higher mortality rates, this risk is neither constant across GA nor as clear-cut as our traditional SGA and LGA definitions suggest. Fixed birthweight cut-offs, such as the 10th and 90th percentiles, remain pragmatic; however, continuous GA-specific risk curves reveal graded, GA-dependent mortality that a single cut-off cannot capture. These fixed thresholds can exaggerate differences at the extremes and mask gradual changes in risk, underscoring the need for a more nuanced assessment. The aim of our study was neither to predict mortality nor to define an optimal cut-off for risk stratification. Its primary value lies in illustrating the continuous, GA-specific effects of birthweight percentile on perinatal mortality. A more nuanced understanding of these associations, and of the limitations of applying absolute thresholds, may help clinicians interpret risk more thoughtfully and guide decisions about the need for additional evaluation or monitoring. Future research should ideally be prospective and focused on other potentially preventable adverse perinatal outcomes, to determine whether clinically relevant cut-offs exist and ultimately improve patient outcomes.

## Supplementary Information

Below is the link to the electronic supplementary material.

Supplementary file1 (PDF 190 kb)

Supplementary file2 (PDF 28 kb)

Supplementary file3 (PDF 428 kb)

Supplementary file4 (PDF 161 kb)

## Data Availability

The data that support the findings of this study are available from the corresponding author upon reasonable request.

## Abbreviations

AGA: Appropriate for gestational age
BW-for-GA: Birthweight for gestational age
GA: Gestational age
LGA: Large for gestational age
SGA: Small for gestational age

## References

[1] Giapros V, Drougia A, Krallis N, Theocharis P, Andronikou S (2012) Morbidity and mortality patterns in small-for-gestational age infants born preterm. J Matern Fetal Neonatal Med 25:153–157. 10.3109/14767058.2011.565837 21463210 10.3109/14767058.2011.565837

[2] Madden JV, Flatley CJ, Kumar S (2018) Term small-for-gestational-age infants from low-risk women are at significantly greater risk of adverse neonatal outcomes. Am J Obstet Gynecol 218:525.e1-525.e9. 10.1016/j.ajog.2018.02.008 29462628 10.1016/j.ajog.2018.02.008

[3] Damhuis SE, Ganzevoort W, Gordijn SJ (2021) Abnormal fetal growth: small for gestational age, fetal growth restriction, large for gestational age: definitions and epidemiology. Obstet Gynecol Clin North Am 48:267–279. 10.1016/j.ogc.2021.02.002 33972065 10.1016/j.ogc.2021.02.002

[4] Hughes MM, Black RE, Katz J (2017) 2500-g low birth weight cutoff: history and implications for future research and policy. Matern Child Health J 21:283–289. 10.1007/s10995-016-2131-9 27449779 10.1007/s10995-016-2131-9 PMC5290050

[5] Battaglia FC, Lubchenco LO (1967) A practical classification of newborn infants by weight and gestational age. J Pediatr 71:159–163. 10.1016/s0022-3476(67)80066-0 6029463 10.1016/s0022-3476(67)80066-0

[6] American Academy of Pediatrics. Committee on fetus and newborn (1967) Nomenclature for duration of gestation, birth weight and intra-uterine growth. Pediatrics 39:935–939. 6026555

[7] Xu H, Simonet F, Luo ZC (2010) Optimal birth weight percentile cut-offs in defining small- or large-for-gestational-age. Acta Paediatr 99:550–555. 10.1111/j.1651-2227.2009.01674.x 20064130 10.1111/j.1651-2227.2009.01674.x

[8] Hoftiezer L, Hukkelhoven CW, Hogeveen M, Straatman HM, van Lingen RA (2016) Defining small-for-gestational-age: prescriptive versus descriptive birthweight standards. Eur J Pediatr 175:1047–1057. 10.1007/s00431-016-2740-8 27255904 10.1007/s00431-016-2740-8

[9] Vasak B, Koenen SV, Koster MP, Hukkelhoven CW, Franx A, Hanson MA, Visser GHA (2015) Human fetal growth is constrained below optimal for perinatal survival. Ultrasound Obstet Gynecol 45:162–167. 10.1002/uog.14644 25092251 10.1002/uog.14644

[10] Kamphof HD, Gordijn SJ, Ganzevoort W, Verfaille V, Offerhaus PM, Franx A, Pajkrt E, de Jonge A, Henrichs J (2022) Associations of severe adverse perinatal outcomes among continuous birth weight percentiles on different birth weight charts: a secondary analysis of a cluster randomized trial. BMC Pregnancy Childbirth 22:375. 10.1186/s12884-022-04680-5 35490210 10.1186/s12884-022-04680-5 PMC9055757

[11] Perined (2018) Perinatale Zorg in Nederland 2016. Perined, Utrecht

[12] Vos AA, Denktaş S, Borsboom GJ, Bonsel GJ, Steegers EA (2015) Differences in perinatal morbidity and mortality on the neighbourhood level in Dutch municipalities: a population based cohort study. BMC Pregnancy Childbirth 2(15):201. 10.1186/s12884-015-0628-7 10.1186/s12884-015-0628-7 PMC4557854 26330115

[13] de Laat MW, Wiegerinck MM, Walther FJ, Boluyt N, Mol BW, van der Post JA, van Lith JM, Offringa M, Nederlandse Vereniging voor Kindergeneeskunde, & Nederlandse Vereniging voor Obstetrie en Gynaecologie (2010) Richtlijn ‘Perinataal beleid bij extreme vroeggeboorte’ [Practice guideline ‘Perinatal management of extremely preterm delivery’]. Ned Tijdschr Geneeskd 154:A2701 21429260

[14] Hoftiezer L, Hof MHP, Dijs-Elsinga J, Hogeveen M, Hukkelhoven CWPM, van Lingen RA (2019) From population reference to national standard: new and improved birthweight charts. Am J Obstet Gynecol 220:383.e1-383.e17. 10.1016/j.ajog.2018.12.023 30576661 10.1016/j.ajog.2018.12.023

[15] Blencowe H, Hug L, Moller AB, You D, Moran AC (2025) Definitions, terminology and standards for reporting of births and deaths in the perinatal period: International Classification of Diseases (ICD-11). Int J Gynaecol Obstet 168:1–9. 10.1002/ijgo.15794 39127912 10.1002/ijgo.15794 PMC11649847

[16] Marra G, Wood SN (2012) Coverage properties of confidence intervals for generalized additive model components. Scand J Stat 39:53–74. 10.1111/j.1467-9469.2011.00760.x

[17] Wood SN (2011) Fast stable restricted maximum likelihood and marginal likelihood estimation of semiparametric generalized linear models. J R Stat Soc Ser B Methodol 73:3–36. 10.1111/j.1467-9868.2010.00749.x

[18] Glinianaia SV, Rankin J, Pearce MS, Parker L, Pless-Mulloli T (2010) Stillbirth and infant mortality in singletons by cause of death, birthweight, gestational age and birthweight-for-gestation, Newcastle upon Tyne 1961–2000. Paediatr Perinat Epidemiol 24:331–342. 10.1111/j.1365-3016.2010.01119.x 20618722 10.1111/j.1365-3016.2010.01119.x

[19] Cole TJ, Hey E, Richmond S (2010) The PREM score: a graphical tool for predicting survival in very preterm births. Arch Dis Child Fetal Neonatal Ed 95:F14–F19. 10.1136/adc.2009.164533 19700396 10.1136/adc.2009.164533

[20] Yu J, Flatley C, Greer RM, Kumar S (2018) Birth-weight centiles and the risk of serious adverse neonatal outcomes at term. J Perinat Med 46:1048–1056. 10.1515/jpm-2017-0176 29257760 10.1515/jpm-2017-0176

[21] Iliodromiti S, Mackay DF, Smith GC, Pell JP, Sattar N, Lawlor DA, Nelson SM (2017) Customised and noncustomised birth weight centiles and prediction of stillbirth and infant mortality and morbidity: a cohort study of 979,912 term singleton pregnancies in Scotland. PLoS Med 14:e1002228. 10.1371/journal.pmed.1002228 28141865 10.1371/journal.pmed.1002228 PMC5283655

[22] Boulet SL, Alexander GR, Salihu HM, Kirby RS, Carlo WA (2006) Fetal growth risk curves: defining levels of fetal growth restriction by neonatal death risk. Am J Obstet Gynecol 195:1571–1577. 10.1016/j.ajog.2006.03.069 16769013 10.1016/j.ajog.2006.03.069

[23] Melamed N, Baschat A, Yinon Y et al (2021) FIGO (International Federation of Gynecology and Obstetrics) initiative on fetal growth: best practice advice for screening, diagnosis, and management of fetal growth restriction. Int J Gynaecol Obstet 152(Suppl 1):3–57. 10.1002/ijgo.13522 33740264 10.1002/ijgo.13522 PMC8252743

[24] Grobman WA, Rice MM, Reddy UM et al (2018) Labor induction versus expectant management in low-risk nulliparous women. N Engl J Med 379:513–523. 10.1056/NEJMoa1800566 30089070 10.1056/NEJMoa1800566 PMC6186292

[25] Atwani R, Saade G, Kawakita T (2025) Impact of the ARRIVE trial on stillbirth rates in nulliparous individuals. Am J Perinatol 42:401–408. 10.1055/s-0044-1789018 39137898 10.1055/s-0044-1789018

[26] Yudkin PL, Wood L, Redman CW (1987) Risk of unexplained stillbirth at different gestational ages. Lancet 1:1192–1194. 10.1016/s0140-6736(87)92154-4 2883499 10.1016/s0140-6736(87)92154-4

[27] Man J, Hutchinson JC, Ashworth M, Heazell AE, Levine S, Sebire NJ (2016) Effects of intrauterine retention and postmortem interval on body weight following intrauterine death: implications for assessment of fetal growth restriction at autopsy. Ultrasound Obstet Gynecol 48:574–578. 10.1002/uog.16018 27781321 10.1002/uog.16018

[28] Modzelewski J, Pokropek A, Jakubiak-Proć M, Muzyka-Placzyńska K, Filipecka-Tyczka D, Kajdy A, Rabijewski M (2022) Large-for-gestational-age or macrosomia as a classifier for risk of adverse perinatal outcome: a retrospective cross-sectional study. J Matern Fetal Neonatal Med 35:5564–5571. 10.1080/14767058.2021.1887127 33602007 10.1080/14767058.2021.1887127

[29] Baer RJ, Rogers EE, Partridge JC, Anderson JG, Morris M, Kuppermann M, Franck LS, Rand L, Jelliffe-Pawlowski LL (2016) Population-based risks of mortality and preterm morbidity by gestational age and birth weight. J Perinatol 36:1008–1013. 10.1038/jp.2016.118 27467566 10.1038/jp.2016.118

[30] Joseph KS, Kramer MS, Allen AC, Mery LS, Platt RW, Wen SW (2001) Implausible birth weight for gestational age. Am J Epidemiol 153:110–113. 10.1093/aje/153.2.110 11159154 10.1093/aje/153.2.110

