# Supplementary material for: Beyond cut-offs: gestational age-specific perinatal mortality across the birthweight-for-gestational-age continuum—a population-based cross-sectional study
Source: Eur J Pediatr. 2026 Jul 15;185(8):579. doi: 10.1007/s00431-026-07233-6 (PMC13373010; doi:10.1007/s00431-026-07233-6)
Supplement: Supplementary file 1 — Supplementary file1 (PDF 190 kb) [file 431_2026_7233_MOESM1_ESM.pdf]

Supplementary File 1. Baseline characteristics of the study population

	**Reference population**		**Excluded records**	
	n	%	n	%
	2,581,816	89.6	299,752	10.4
**Sex assigned at birth**				
Male	1,317,381	51.0	159,933	53.8
Female	1,264,435	49.0	137,171	46.2
Missing	0	0.0	2,648	0.9
**Parity**				
Nulliparous	1,173,316	45.4	148,909	49.7
Multiparous	1,408,362	54.6	150,745	50.3
Missing	138	0.0	98	0.0
**Socioeconomic status**[a]				
Very low	686,235	26.9	82,069	27.9
Low	479,679	18.8	55,237	18.8
Average	440,669	17.3	50,409	17.1
High	427,712	16.7	48,838	16.6
Very high	519,437	20.3	57,883	19.7
Missing	28,084	1.1	5,316	1.8
**Maternal age, years**				
<18	8,765	0.3	1,310	0.4
18-35	2,189,552	84.8	245,520	82.0
≥35	383,203	14.8	52,609	17.6
Missing	296	0.0	313	0.1
**Gestational age**				
<28 weeks	5,623	0.2	16,829	5.6
28-<32 weeks	13,716	0.5	9,753	3.3
32-<37 weeks	127,375	4.9	52,922	17.7
≥37 weeks	2,435,102	94.3	185,455	61.9
Mean, SD (days)	277.0	12.5	263.4	33.8
Missing	0	0.0	34,793	11.6
**Birthweight, grams**				
<1000	6,457	0.3	18,368	6.1
1000-<2000	31,539	1.2	27,079	9.0
2000-<3000	419,164	16.2	93,782	31.3
3000-<4000	1,742,000	67.5	119,248	39.8
≥4000	382,656	14.8	37,869	12.6
Mean, SD	3,446.0	559.1	2,948.6	1026.9
Missing	0	0.0	3,406	1.1
**Place of delivery**				
Home delivery	543,018	21.2	17,631	6.0
Midwife-led in-hospital delivery	328,769	12.8	26,442	9.0
Hospital delivery	1,692,481	66.0	248,676	84.9
Missing	17,548	0.7	7,003	2.3

[a]Socioeconomic status was determined at the area level using the educational level, employment rate, and income level of the maternal residential postal code area.

Beyond cut-offs: gestational age-specific perinatal mortality across the birthweight-for-gestational-age continuum – a population-based cross-sectional study. European Journal of Pediatrics. Liset Hoftiezer, Michel H.P. Hof, Richard A. van Lingen, Chantal W.P.M. Hukkelhoven, Marije Hogeveen.

Corresponding author: Liset Hoftiezer, Department of Pediatrics, Slingeland Hospital, Doetinchem, The Netherlands [liset.hoftiezer2@slingeland.nl]
