# Supplementary figures and images for: Beyond cut-offs: gestational age-specific perinatal mortality across the birthweight-for-gestational-age continuum—a population-based cross-sectional study
Source: Eur J Pediatr. 2026 Jul 15;185(8):579. doi: 10.1007/s00431-026-07233-6 (PMC13373010; doi:10.1007/s00431-026-07233-6)

Intercept  $\alpha$ 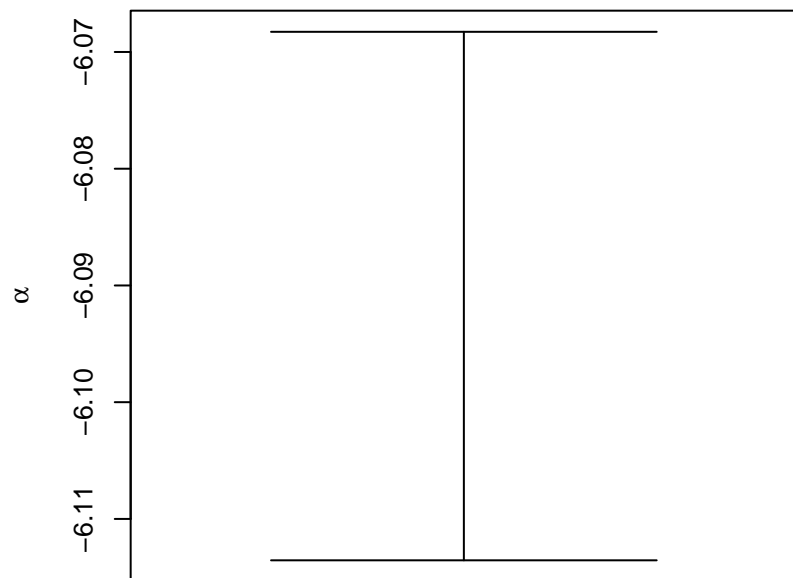Main effect  $f_1(x)$ 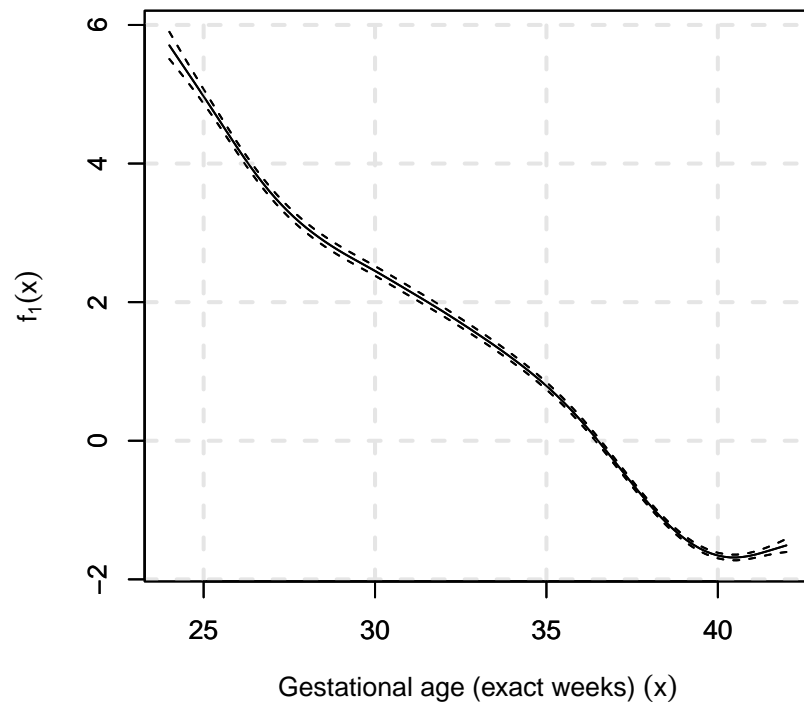Main effect  $f_2(z)$ 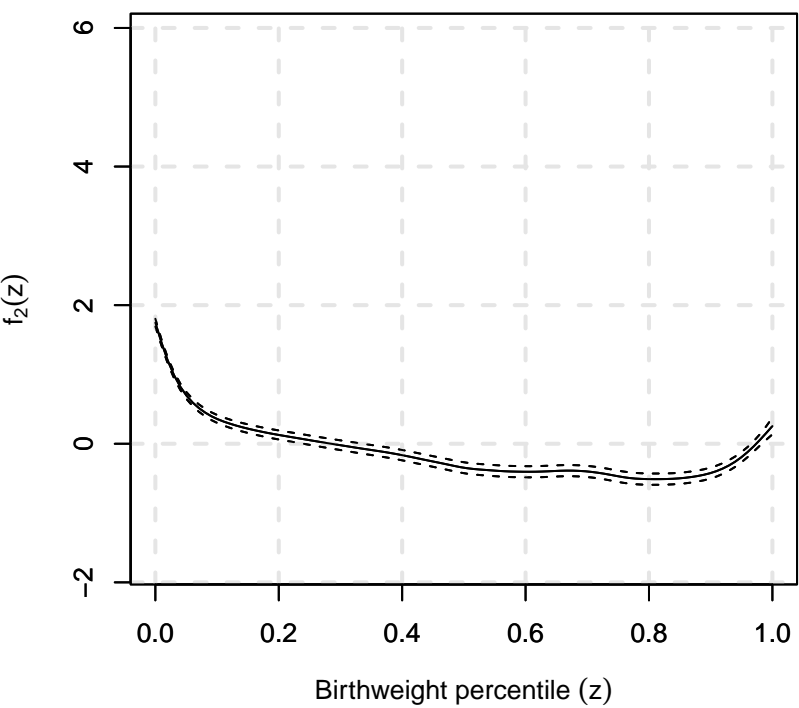Interaction  $f_3(x, z)$ 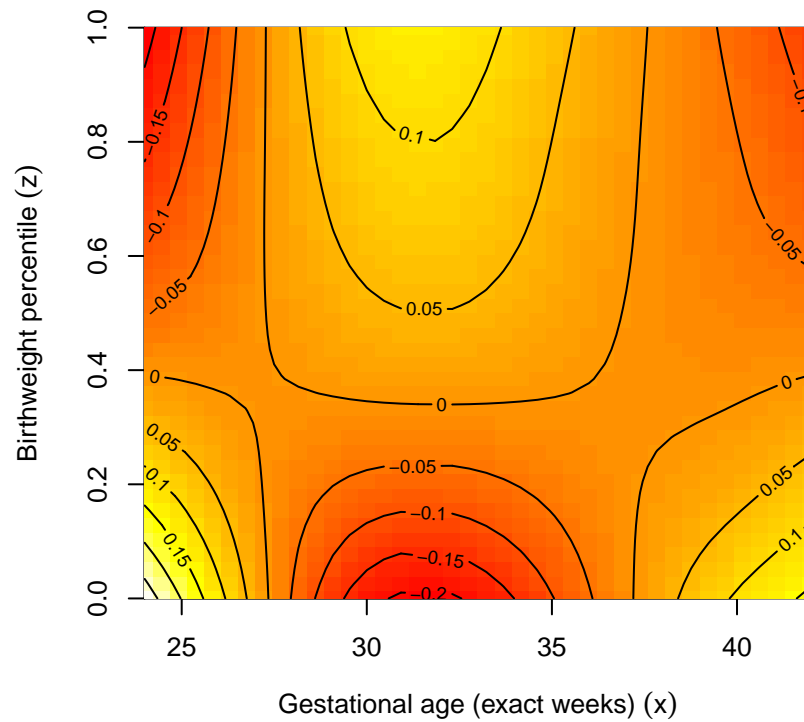

Supplement: Supplementary file 2 — Supplementary file2 (PDF 28 kb) [file 431_2026_7233_MOESM2_ESM.pdf]
