# Supplementary material for: Beyond cut-offs: gestational age-specific perinatal mortality across the birthweight-for-gestational-age continuum—a population-based cross-sectional study
Source: Eur J Pediatr. 2026 Jul 15;185(8):579. doi: 10.1007/s00431-026-07233-6 (PMC13373010; doi:10.1007/s00431-026-07233-6)
Supplement: Supplementary file 3 — Supplementary file3 (PDF 428 kb) [file 431_2026_7233_MOESM3_ESM.pdf]

Supplementary File 3.

<u>Sensitivity analyses</u>

After the exclusion of antepartum stillbirths, we modelled the association between perinatal mortality (i.e., intrapartum fetals deaths plus early neonatal deaths) with the following logistic regression model:

$$\log\left[\frac{\Pr(\text{perinatal mortality})}{1-\Pr(\text{perinatal mortality})}\right] = \alpha + f_{1,\lambda_1}(\text{gestational age}) + f_{2,\lambda_2}(\text{birthweight percentile}) + f_{3,\lambda_3}(\text{gestational age, birthweight percentile})$$

Fig. 1 Main effects of explanatory variables

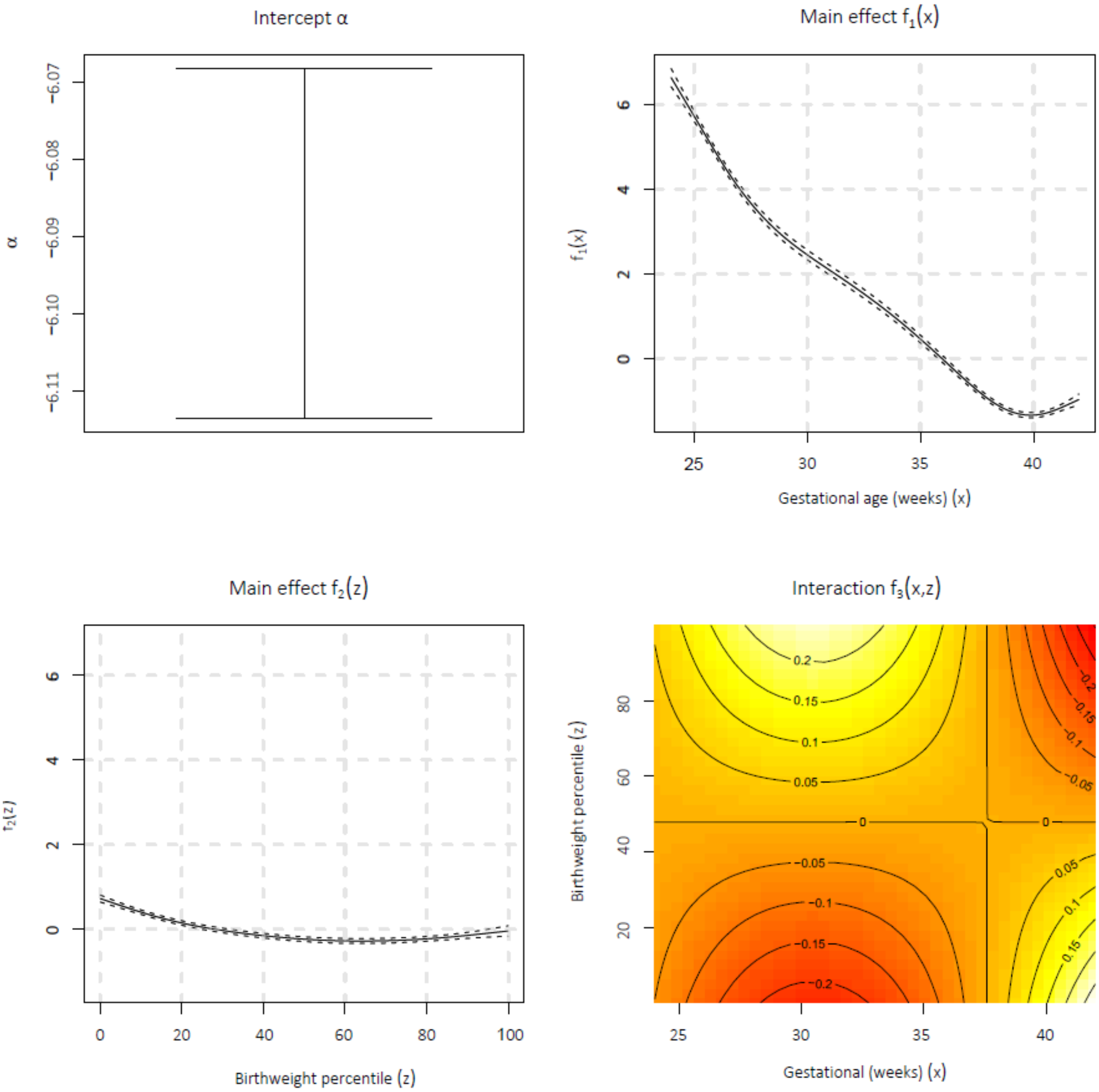

Fig. 2 Predicted mortality probability (gestational age 24-27 weeks)

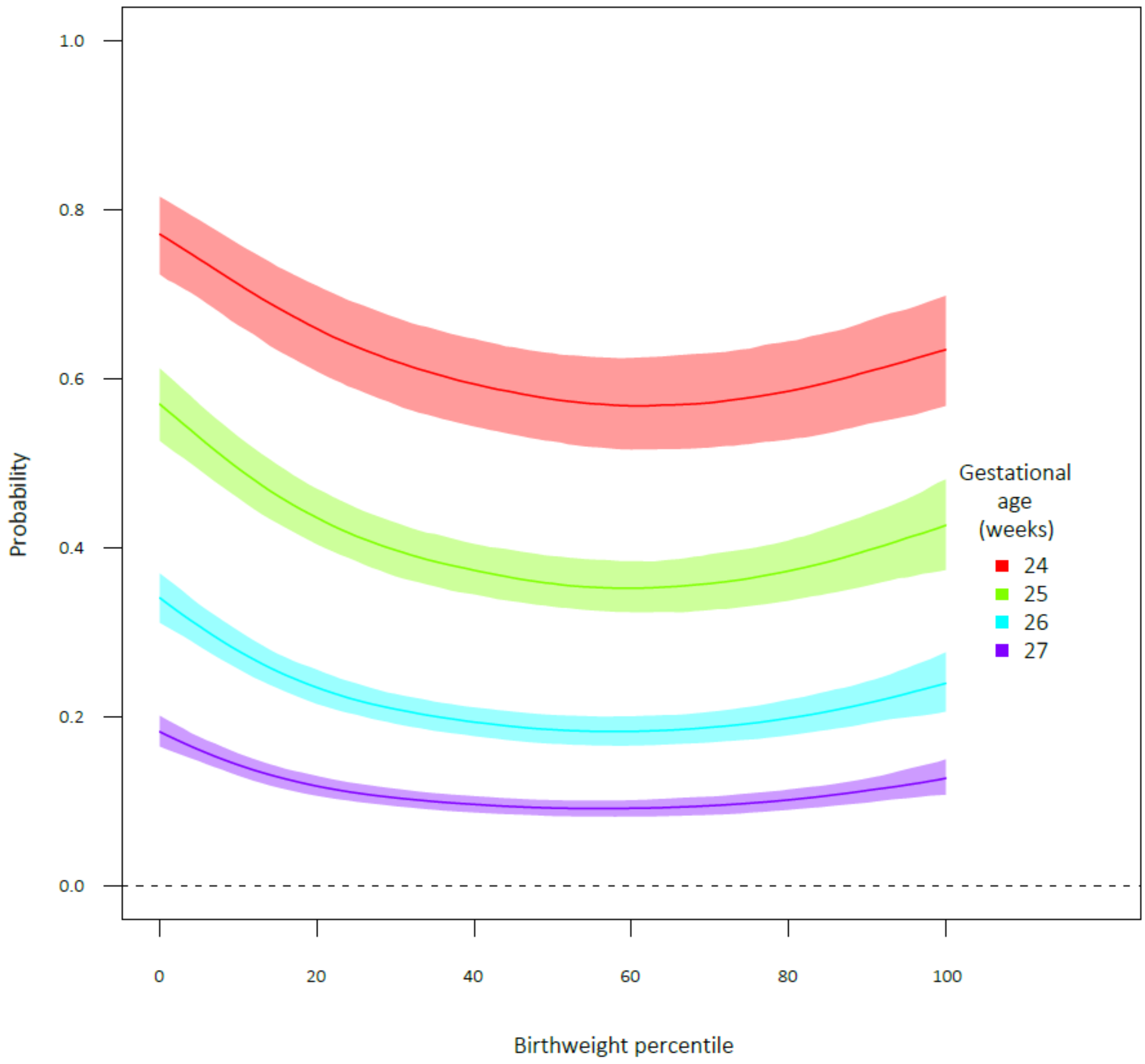

Fig. 3 Predicted mortality probability (gestational age 28-31 weeks)

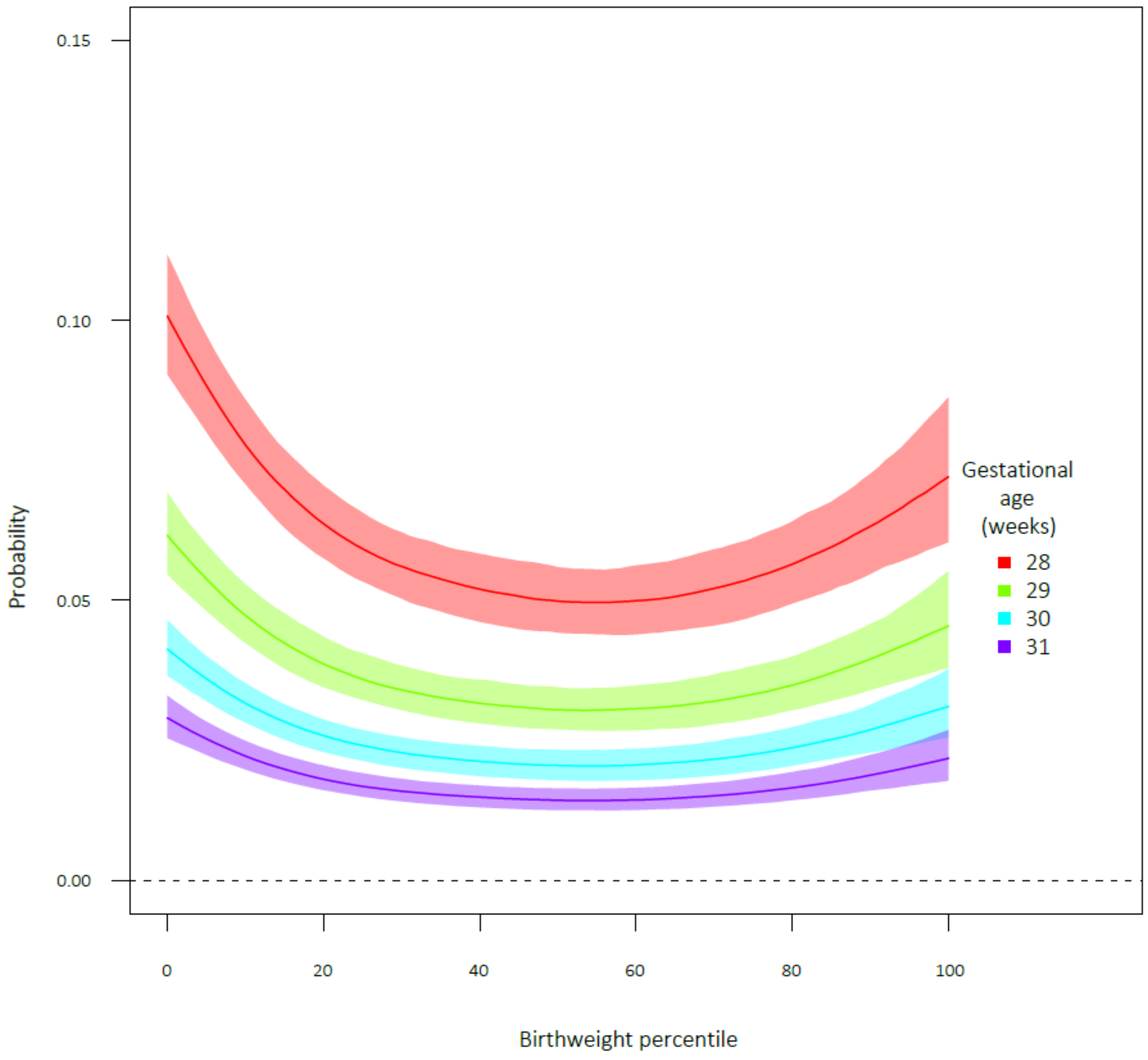

Fig. 4 Predicted mortality probability (gestational age 32-36 weeks)

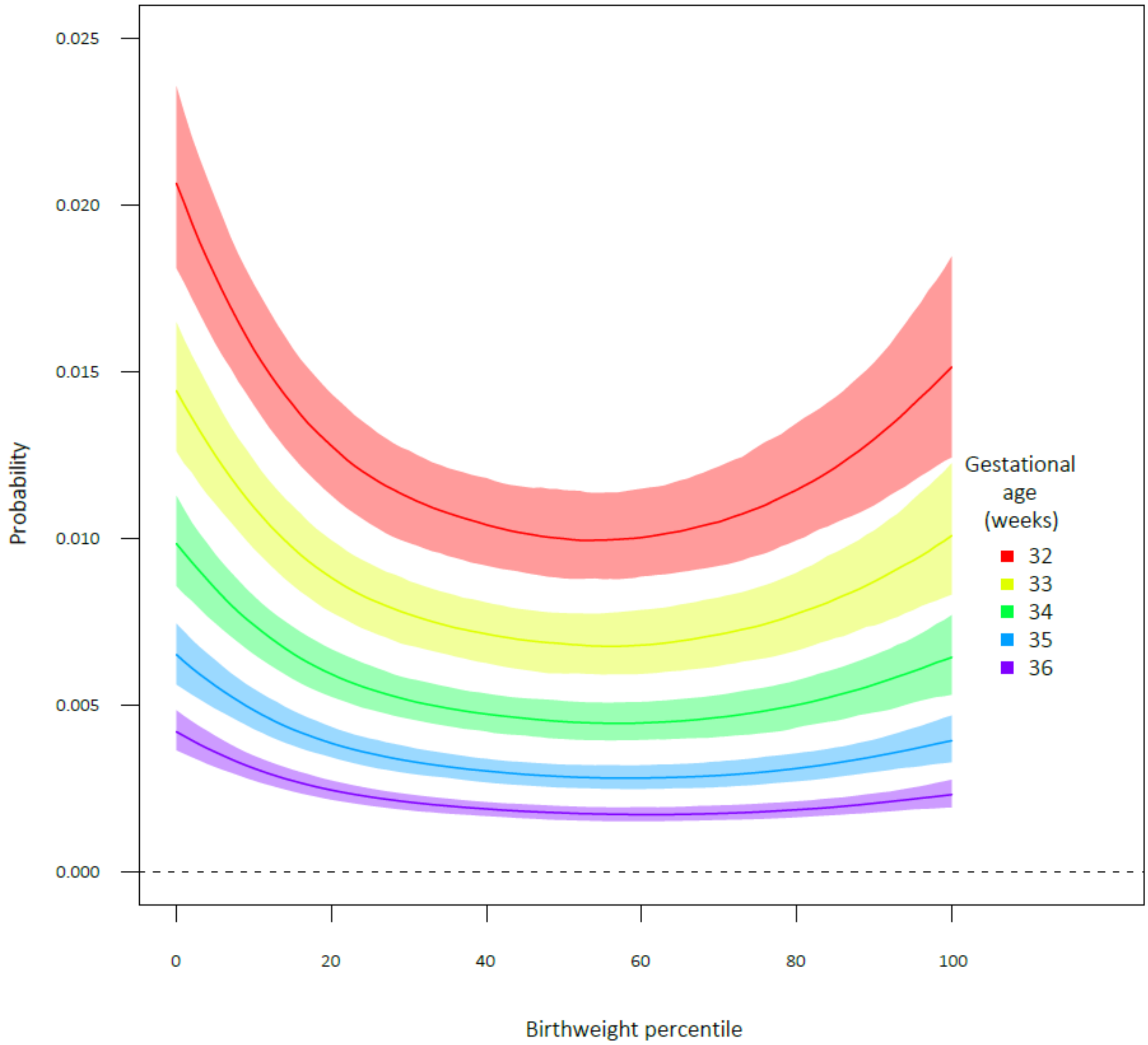

Fig. 5 Predicted mortality probability (gestational age 37-39 weeks)

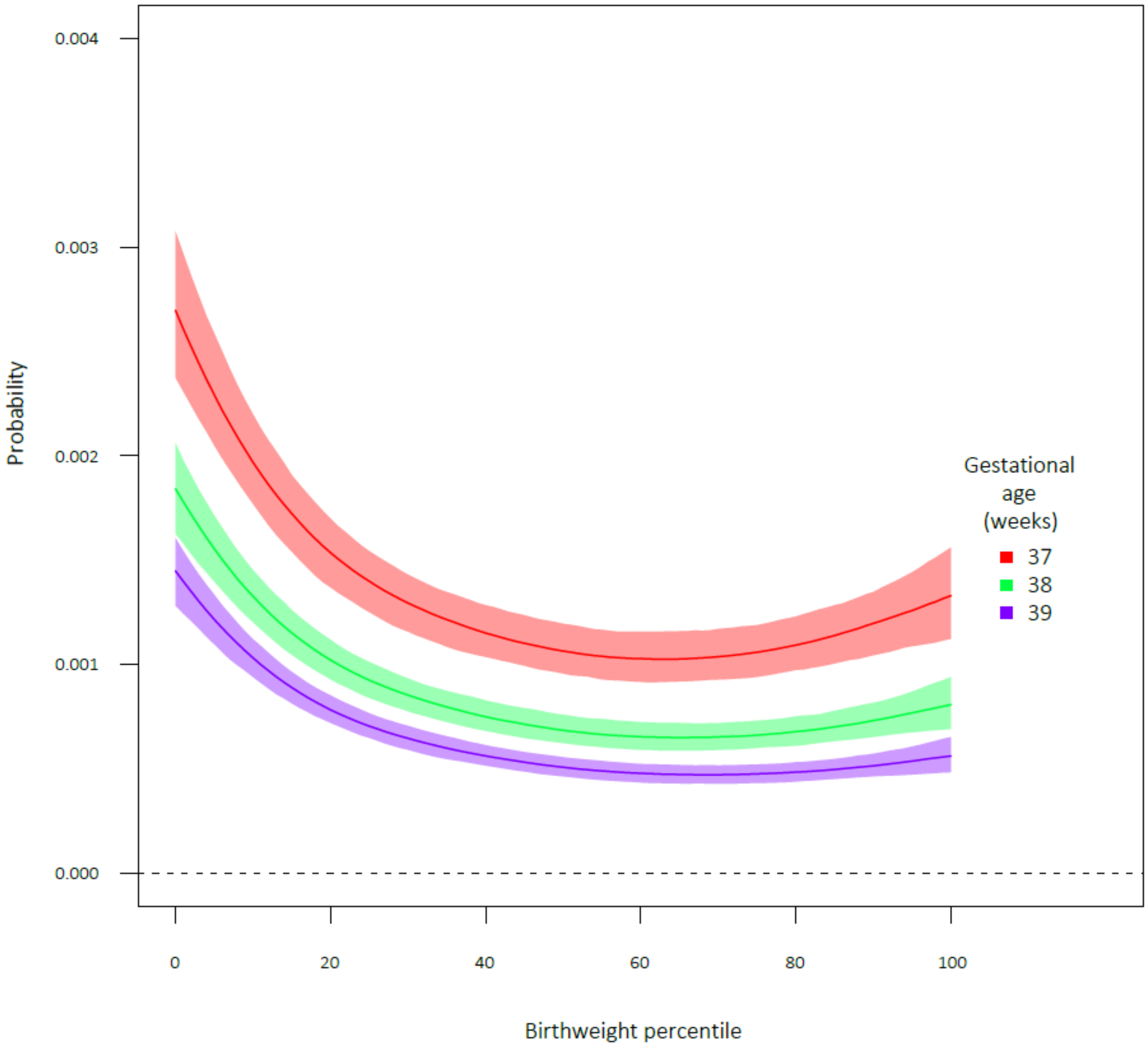

Fig. 6 Predicted mortality probability (gestational age 40-42 weeks)

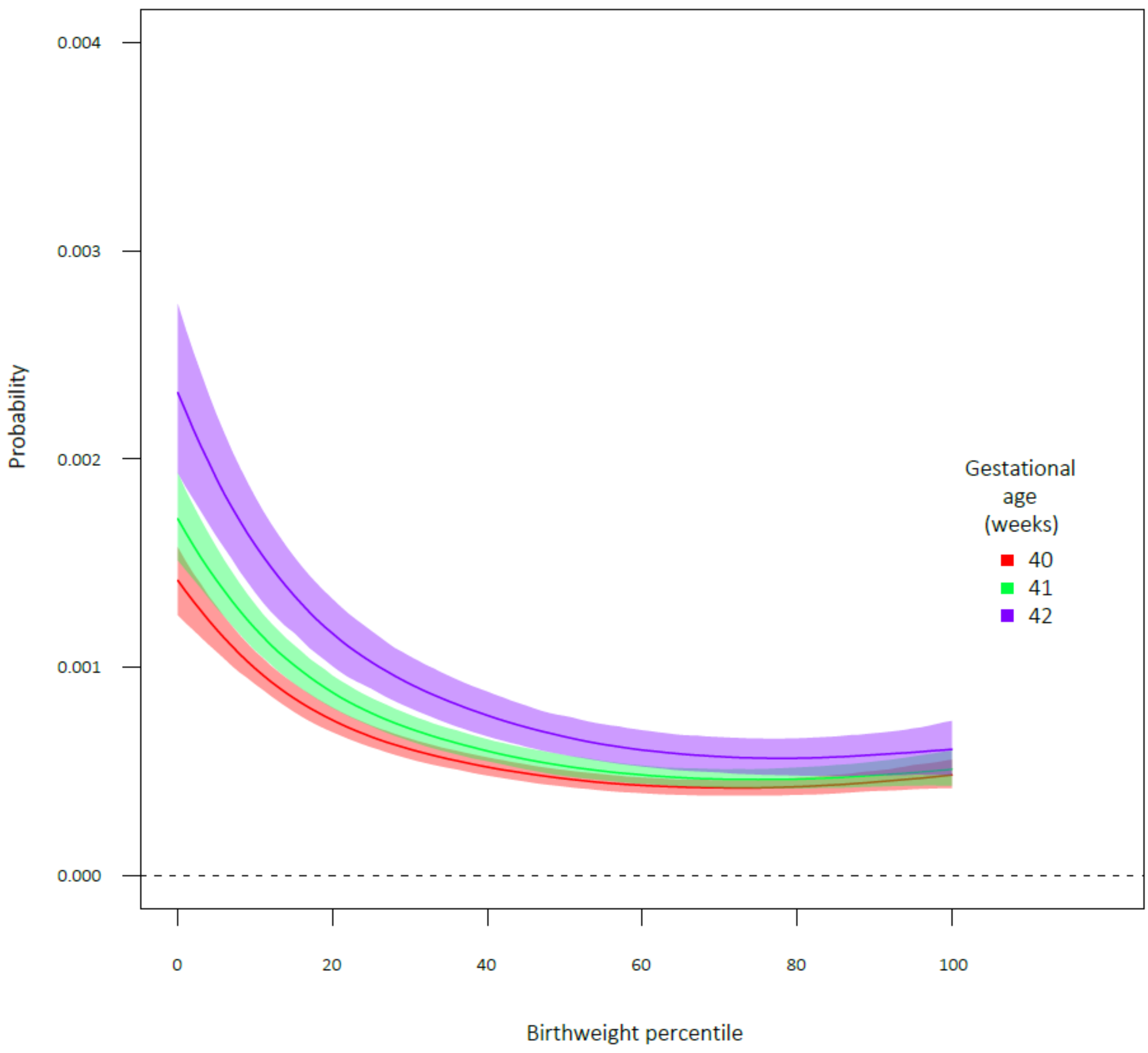

Beyond cut-offs: gestational age-specific perinatal mortality across the birthweight-for-gestational-age continuum – a population-based cross-sectional study. European Journal of Pediatrics. Liset Hoftiezer, Michel H.P. Hof, Richard A. van Lingen, Chantal W.P.M. Hukkelhoven, Marije Hogeveen.

Corresponding author:

Liset Hoftiezer, Department of Pediatrics, Slingeland Hospital, Doetinchem, The Netherlands [liset.hoftiezer2@slingeland.nl]
